# Tetra­aqua­bis[4-(imidazol-1-yl-κ*N* ^3^)benzoato]manganese(II)

**DOI:** 10.1107/S1600536810012638

**Published:** 2010-04-10

**Authors:** Lei Zhu, Dabin Wang, Hongyan Xu

**Affiliations:** a Wuhan Institute of Technology, School of Chemical Engineering and Pharmacy, Hubei 430073, People’s Republic of China; b Fujian Institute of Research on the Structure of Matter, Chinese Academy of Sciences, Fuzhou 350002, People’s Republic of China

## Abstract

In the title compound, [Mn(C_10_H_7_N_2_O_2_)_2_(H_2_O)_4_], the Mn^II^ atom, lying on an inversion center, has an octa­hedral environment with four coordinated water mol­ecules in the equatorial plane and two N atoms from two 4-(imidazol-1-yl)benzoate ligands at the axial sites. The complex mol­ecules are connected into a three-dimensional network by extensive hydrogen bonds between the water mol­ecules and the carboxyl­ate O atoms.

## Related literature

For the good coordination ability and diverse coordination modes of ligands containing imidazole and carboxyl­ate groups, see: Fan *et al.* (2004 ▶); Sun *et al.* (2005 ▶). For the construction of metal–organic frameworks using ligands based on imidazolyl and carboxyl­ate groups as building blocks, see: Carlucci *et al.* (2008 ▶); Zhang *et al.* (2007 ▶).
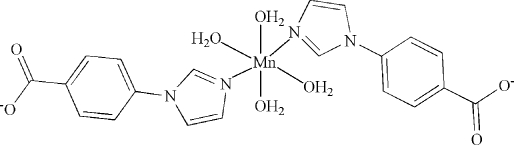


## Experimental

### 

#### Crystal data

[Mn(C_10_H_7_N_2_O_2_)_2_(H_2_O)_4_]
*M* *_r_* = 501.36
Monoclinic, 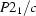

*a* = 12.278 (12) Å
*b* = 11.026 (11) Å
*c* = 7.978 (7) Å
β = 96.91 (2)°
*V* = 1072.2 (18) Å^3^
*Z* = 2
Mo *K*α radiation
μ = 0.67 mm^−1^
*T* = 293 K
0.28 × 0.14 × 0.10 mm

#### Data collection

Bruker SMART APEX CCD diffractometer
Absorption correction: multi-scan (*SADABS*; Sheldrick, 1996 ▶) *T*_min_ = 0.536, *T*_max_ = 1.000
7972 measured reflections
2444 independent reflections
2131 reflections with *I* > 2σ(*I*)
*R*_int_ = 0.023

#### Refinement

*R*[*F* ^2^ > 2σ(*F* ^2^)] = 0.032
*wR*(*F* ^2^) = 0.113
*S* = 0.91
2444 reflections
195 parameters
All H-atom parameters refined
Δρ_max_ = 0.36 e Å^−3^
Δρ_min_ = −0.23 e Å^−3^

### 

Data collection: *SMART* (Bruker, 2007 ▶); cell refinement: *SAINT* (Bruker, 2007 ▶); data reduction: *SAINT*; program(s) used to solve structure: *SHELXS97* (Sheldrick, 2008 ▶); program(s) used to refine structure: *SHELXL97* (Sheldrick, 2008 ▶); molecular graphics: *SHELXTL* (Sheldrick, 2008 ▶); software used to prepare material for publication: *SHELXTL*.

## Supplementary Material

Crystal structure: contains datablocks I, global. DOI: 10.1107/S1600536810012638/hy2292sup1.cif

Structure factors: contains datablocks I. DOI: 10.1107/S1600536810012638/hy2292Isup2.hkl

Additional supplementary materials:  crystallographic information; 3D view; checkCIF report

## Figures and Tables

**Table 1:** Hydrogen-bond geometry (Å, °)

*D*—H⋯*A*	*D*—H	H⋯*A*	*D*⋯*A*	*D*—H⋯*A*
O3—H13⋯O1^i^	0.85 (2)	2.02 (2)	2.837 (3)	161 (2)
O3—H14⋯O2^ii^	0.79 (3)	1.90 (3)	2.688 (3)	179 (2)
O4—H11⋯O1^iii^	0.90 (2)	1.82 (2)	2.702 (3)	168 (2)
O4—H12⋯O1^i^	0.87 (2)	1.86 (2)	2.694 (3)	158.9 (19)

Symmetry codes: (i) 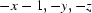; (ii) 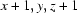; (iii) 
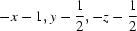
.

**Fig. 1.**
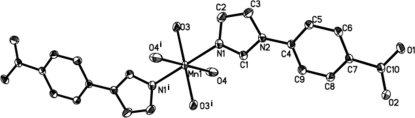
The molecular structure of the title compound, with the 50% probability displacement ellipsoids. H atoms have been omitted for clarity. [Symmetry code: (i) -x, -y, -z.]

**Fig. 2.**
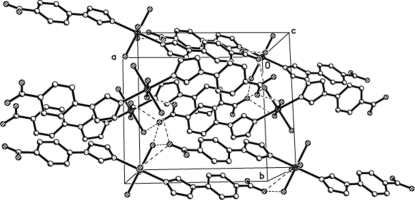
The crystal packing of the title compound, showing O—H···O hydrogen bonds (dashed lines). H atoms not involved in hydrogen bonding have been omitted.

## supplementary crystallographic information

### Comment

Over past few years, considerable effort was paied in the study of metal-organic frameworks (MOFs) owing to their intriguing structural diversity and potential application in adsorption, molecular recognition, catalysis and maganetism. The field of molecular magnets has attracted great interest from different horizons for many years. In this context, the ligand containing imidazole and carboxylate groups is of special interest due to its good coordination ability and diverse coordination modes (Fan *et al.*, 2004; Sun *et al.*, 2005). However, the reports of ligands based on imidazole and carboxylate groups as building blocks for the construction of MOFs (Carlucci *et al.*, 2008; Zhang *et al.*, 2007) are still rare. In this paper, we report the synthesis and structural characterzation of the title compound.

As shown in Fig. 1, the molecular structure of the title compound is a momonuclear Mn^II^ complex and the dihedral angle between the imidazolyl ring and the benzene ring of the 4-(imidazol-1-yl)benzoate is 6.3 (2)°. The Mn^II^ ion is coordinated by four water molecules and two N atoms from two different 4-(imidazol-1-yl)benzoate ligands, forming a distorted octahedral coordination environment. The Mn—N and Mn—O bond distances are 2.238 (2) Å and 2.149 (2) and 2.189 (3) Å, respectively. The related hydrogen-bonding geometry is given in Table 1. A l l values involved with hydrogen bonds fall in a normal range. The intermolecular O—H···O hydrogen-bonding interactions between the coordinated water molecules and carboxylate O atoms of 4-(imidazol-1-yl)benzoate ligands lead to the formation of a three-dimensional network structure as shown in Fig. 2.

### Experimental

A 10 ml aqueous solution of 4-(imidazole-1-yl)benzoic acid (0.038 g, 0.20 mmol) was slowly added into the manganese(II) perchlorate (0.663 g, 0.30 mmol) solution in methanol (10 ml). The mixed solution was stirred for 20 min and then HClO_4_ solution was added dropwise with constant stirring until the mixed solution was clear. The resulting solution was filtered and the slow evaporation of filtrate in air gave rise to the desirable products, which were subsequently washed twice with Et_2_O (yield 38%).

### Refinement

All H atoms were located in a difference Fourier map and refined isotropically.

### Crystal data

**Table:**

[Mn(C_10_H_7_N_2_O_2_)_2_(H_2_O)_4_]	*F*(000) = 518
*M**_r_* = 501.36	*D*_x_ = 1.553 Mg m^−^^3^
Monoclinic, *P*2_1_/*c*	Mo *K*α radiation, λ = 0.71073 Å
Hall symbol: -P 2ybc	Cell parameters from 3126 reflections
*a* = 12.278 (12) Å	θ = 3.3–27.5°
*b* = 11.026 (11) Å	µ = 0.67 mm^−^^1^
*c* = 7.978 (7) Å	*T* = 293 K
β = 96.91 (2)°	Prism, yellow
*V* = 1072.2 (18) Å^3^	0.28 × 0.14 × 0.10 mm
*Z* = 2	

### Data collection

**Table:**

Bruker SMART APEX CCD diffractometer	2444 independent reflections
Radiation source: fine-focus sealed tube	2131 reflections with *I* > 2σ(*I*)
graphite	*R*_int_ = 0.023
φ and ω scans	θ_max_ = 27.5°, θ_min_ = 2.5°
Absorption correction: multi-scan (*SADABS*; Sheldrick, 1996)	*h* = −15→15
*T*_min_ = 0.536, *T*_max_ = 1.000	*k* = −14→13
7972 measured reflections	*l* = −10→10

### Refinement

**Table:**

Refinement on *F*^2^	Primary atom site location: structure-invariant direct methods
Least-squares matrix: full	Secondary atom site location: difference Fourier map
*R*[*F*^2^ > 2σ(*F*^2^)] = 0.032	Hydrogen site location: inferred from neighbouring sites
*wR*(*F*^2^) = 0.113	All H-atom parameters refined
*S* = 0.91	*w* = 1/[σ^2^(*F*_o_^2^) + (0.1*P*)^2^] where *P* = (*F*_o_^2^ + 2*F*_c_^2^)/3
2444 reflections	(Δ/σ)_max_ < 0.001
195 parameters	Δρ_max_ = 0.36 e Å^−^^3^
0 restraints	Δρ_min_ = −0.23 e Å^−^^3^

### Fractional atomic coordinates and isotropic or equivalent isotropic displacement parameters (Å^2^)

**Table:**

	*x*	*y*	*z*	*U*_iso_*/*U*_eq_	
Mn1	0.0000	0.0000	0.0000	0.02675 (15)	
O1	−0.85062 (8)	0.13841 (11)	−0.33828 (13)	0.0376 (3)	
O2	−0.77609 (10)	0.03929 (15)	−0.53834 (16)	0.0491 (3)	
O3	0.02777 (12)	0.01354 (13)	0.27549 (16)	0.0452 (3)	
O4	−0.07938 (14)	−0.17041 (13)	0.03407 (19)	0.0636 (5)	
N1	−0.16150 (10)	0.09039 (13)	0.01711 (16)	0.0364 (3)	
N2	−0.33890 (9)	0.12493 (11)	−0.04316 (14)	0.0289 (3)	
C1	−0.25122 (12)	0.06006 (16)	−0.0792 (2)	0.0383 (4)	
C2	−0.19202 (15)	0.17902 (19)	0.1215 (3)	0.0523 (5)	
C3	−0.30063 (15)	0.20096 (19)	0.0867 (3)	0.0550 (5)	
C4	−0.44787 (10)	0.11462 (12)	−0.12551 (16)	0.0259 (3)	
C5	−0.52854 (12)	0.19507 (14)	−0.08855 (19)	0.0325 (3)	
C6	−0.63403 (12)	0.18533 (14)	−0.1733 (2)	0.0332 (3)	
C7	−0.65917 (11)	0.09772 (13)	−0.29617 (17)	0.0265 (3)	
C8	−0.57766 (14)	0.01717 (15)	−0.3290 (2)	0.0347 (4)	
C9	−0.47314 (14)	0.02432 (16)	−0.2445 (2)	0.0372 (4)	
C10	−0.77070 (11)	0.09005 (14)	−0.39869 (17)	0.0301 (3)	
H1	−0.254 (3)	0.0002 (18)	−0.158 (4)	0.067 (8)*	
H2	−0.139 (2)	0.212 (2)	0.210 (3)	0.068 (7)*	
H3	−0.347 (2)	0.254 (2)	0.135 (3)	0.070 (7)*	
H5	−0.5108 (14)	0.263 (2)	−0.006 (2)	0.047 (6)*	
H6	−0.6944 (15)	0.2366 (18)	−0.149 (2)	0.039 (5)*	
H8	−0.5940 (17)	−0.048 (2)	−0.418 (3)	0.052 (5)*	
H9	−0.4164 (18)	−0.036 (2)	−0.264 (2)	0.048 (5)*	
H11	−0.0941 (19)	−0.239 (2)	−0.025 (3)	0.065 (6)*	
H12	−0.1116 (17)	−0.1765 (18)	0.126 (3)	0.047 (5)*	
H13	−0.016 (2)	−0.035 (2)	0.316 (3)	0.050 (6)*	
H14	0.086 (2)	0.021 (2)	0.330 (3)	0.060 (7)*	

### Atomic displacement parameters (Å^2^)

**Table:**

	*U*^11^	*U*^22^	*U*^33^	*U*^12^	*U*^13^	*U*^23^
Mn1	0.0172 (2)	0.0347 (2)	0.0279 (2)	−0.00251 (9)	0.00068 (13)	−0.00317 (10)
O1	0.0226 (5)	0.0550 (7)	0.0358 (6)	0.0086 (4)	0.0055 (4)	0.0051 (5)
O2	0.0278 (6)	0.0822 (9)	0.0354 (6)	0.0050 (6)	−0.0040 (5)	−0.0161 (6)
O3	0.0302 (7)	0.0748 (9)	0.0294 (7)	−0.0122 (6)	−0.0018 (5)	−0.0016 (5)
O4	0.0892 (11)	0.0500 (8)	0.0604 (9)	−0.0349 (8)	0.0443 (9)	−0.0225 (7)
N1	0.0238 (6)	0.0475 (8)	0.0367 (6)	0.0026 (5)	−0.0010 (5)	−0.0049 (6)
N2	0.0225 (6)	0.0352 (6)	0.0286 (6)	0.0042 (4)	0.0020 (4)	−0.0011 (5)
C1	0.0229 (7)	0.0500 (10)	0.0413 (8)	0.0063 (6)	0.0008 (6)	−0.0125 (7)
C2	0.0345 (9)	0.0640 (12)	0.0547 (11)	0.0065 (8)	−0.0093 (8)	−0.0253 (9)
C3	0.0340 (9)	0.0664 (12)	0.0613 (12)	0.0119 (8)	−0.0084 (8)	−0.0349 (10)
C4	0.0208 (6)	0.0311 (7)	0.0258 (6)	0.0010 (5)	0.0021 (5)	0.0016 (5)
C5	0.0268 (7)	0.0338 (8)	0.0365 (7)	0.0020 (5)	0.0028 (6)	−0.0087 (6)
C6	0.0240 (6)	0.0356 (8)	0.0404 (8)	0.0066 (5)	0.0062 (6)	−0.0035 (6)
C7	0.0216 (6)	0.0308 (7)	0.0274 (6)	0.0006 (5)	0.0045 (5)	0.0040 (5)
C8	0.0274 (8)	0.0400 (8)	0.0355 (9)	0.0045 (6)	−0.0007 (7)	−0.0098 (6)
C9	0.0268 (8)	0.0413 (8)	0.0425 (9)	0.0105 (6)	0.0004 (7)	−0.0116 (7)
C10	0.0233 (6)	0.0388 (8)	0.0285 (7)	0.0005 (5)	0.0037 (5)	0.0072 (6)

### Geometric parameters (Å, °)

**Table:**

Mn1—O4	2.149 (2)	C2—C3	1.351 (3)
Mn1—O3	2.188 (2)	C2—H2	0.97 (2)
Mn1—N1	2.238 (2)	C3—H3	0.93 (3)
O1—C10	1.2623 (19)	C4—C9	1.385 (2)
O2—C10	1.242 (2)	C4—C5	1.387 (2)
O3—H13	0.85 (2)	C5—C6	1.391 (2)
O3—H14	0.79 (3)	C5—H5	1.00 (2)
O4—H11	0.90 (2)	C6—C7	1.385 (2)
O4—H12	0.87 (2)	C6—H6	0.970 (19)
N1—C1	1.308 (2)	C7—C8	1.387 (2)
N1—C2	1.366 (2)	C7—C10	1.511 (2)
N2—C1	1.352 (2)	C8—C9	1.378 (3)
N2—C3	1.371 (2)	C8—H8	1.02 (2)
N2—C4	1.422 (2)	C9—H9	0.99 (2)
C1—H1	0.91 (2)		
			
O4—Mn1—O4^i^	180.00 (9)	N2—C1—H1	125 (2)
O4—Mn1—O3	87.18 (6)	C3—C2—N1	109.84 (16)
O4^i^—Mn1—O3	92.82 (6)	C3—C2—H2	130.1 (14)
O4—Mn1—O3^i^	92.82 (6)	N1—C2—H2	119.9 (14)
O4^i^—Mn1—O3^i^	87.18 (6)	C2—C3—N2	106.62 (16)
O3—Mn1—O3^i^	180.00 (8)	C2—C3—H3	131.2 (15)
O4—Mn1—N1^i^	92.12 (9)	N2—C3—H3	122.2 (15)
O4^i^—Mn1—N1^i^	87.88 (9)	C9—C4—C5	119.91 (14)
O3—Mn1—N1^i^	93.41 (6)	C9—C4—N2	119.65 (12)
O3^i^—Mn1—N1^i^	86.59 (6)	C5—C4—N2	120.44 (13)
O4—Mn1—N1	87.88 (9)	C4—C5—C6	119.61 (14)
O4^i^—Mn1—N1	92.12 (9)	C4—C5—H5	120.7 (10)
O3—Mn1—N1	86.59 (6)	C6—C5—H5	119.6 (10)
O3^i^—Mn1—N1	93.41 (6)	C7—C6—C5	120.87 (13)
N1^i^—Mn1—N1	180.00 (9)	C7—C6—H6	115.9 (11)
Mn1—O3—H13	108.2 (16)	C5—C6—H6	123.2 (11)
Mn1—O3—H14	125.7 (18)	C6—C7—C8	118.45 (14)
H13—O3—H14	115 (2)	C6—C7—C10	122.20 (13)
Mn1—O4—H11	137.2 (15)	C8—C7—C10	119.32 (14)
Mn1—O4—H12	115.5 (13)	C9—C8—C7	121.45 (15)
H11—O4—H12	107.2 (18)	C9—C8—H8	118.5 (12)
C1—N1—C2	105.52 (14)	C7—C8—H8	120.1 (12)
C1—N1—Mn1	122.68 (12)	C8—C9—C4	119.67 (14)
C2—N1—Mn1	131.77 (11)	C8—C9—H9	121.2 (13)
C1—N2—C3	105.95 (14)	C4—C9—H9	119.1 (13)
C1—N2—C4	126.05 (13)	O2—C10—O1	124.98 (13)
C3—N2—C4	128.00 (13)	O2—C10—C7	117.26 (13)
N1—C1—N2	112.07 (15)	O1—C10—C7	117.73 (14)
N1—C1—H1	123 (2)		

Symmetry codes: (i) −*x*, −*y*, −*z*.

### Hydrogen-bond geometry (Å, °)

**Table:**

*D*—H···*A*	*D*—H	H···*A*	*D*···*A*	*D*—H···*A*
O3—H13···O1^ii^	0.85 (2)	2.02 (2)	2.837 (3)	161 (2)
O3—H14···O2^iii^	0.79 (3)	1.90 (3)	2.688 (3)	179 (2)
O4—H11···O1^iv^	0.90 (2)	1.82 (2)	2.702 (3)	168 (2)
O4—H12···O1^ii^	0.87 (2)	1.86 (2)	2.694 (3)	158.9 (19)

Symmetry codes: (ii) −*x*−1, −*y*, −*z*; (iii) *x*+1, *y*, *z*+1; (iv) −*x*−1, *y*−1/2, −*z*−1/2.
